# Growth patterns in children with spinal muscular atrophy

**DOI:** 10.1186/s13023-021-02015-9

**Published:** 2021-09-04

**Authors:** Ramona De Amicis, Giovanni Baranello, Andrea Foppiani, Alessandro Leone, Alberto Battezzati, Giorgio Bedogni, Simone Ravella, Ester Giaquinto, Chiara Mastella, Caterina Agosto, Enrico Bertini, Adele D’Amico, Marina Pedemonte, Claudio Bruno, Jonathan C. Wells, Mary Fewtrell, Simona Bertoli

**Affiliations:** 1 International Center for the Assessment of Nutritional Status (ICANS), Department of Food, Environmental and Nutritional Sciences (DeFENS), University of Milan, Via Sandro Botticelli 21, 20133 Milan, Italy; 2 UO Neurologia dello Sviluppo, Fondazione IRCCS Istituto Neurologico Carlo Besta, Milan, Italy; 3 The Dubowitz Neuromuscular Centre, UCL NIHR GOSH Biomedical Research Centre, UCL Great Ormond Street Institute of Child Health, London, UK; 4 Dietetic and Nutrition Center, M. Bufalini Hospital, Cesena, Italy; 5 SAPRE-UONPIA, Fondazione IRCCS Ca’ Granda Ospedale Maggiore Policlinico, Milan, Italy; 6 Dipartimento di Salute della Donna e del Bambino, Università di Padova, Padua, Italy; 7 Unità di Malattie Neuromuscolari e Neurodegenerative, Laboratorio di Medicina Molecolare, Dipartimento di Neuroscienze e Neuroriabilitazione, IRCCS Ospedale Pediatrico Bambino Gesù, Rome, Italy; 8 Pediatric Neurology and Muscle Disease Unit, IRCCS Istituto Giannina Gaslini, Genoa, Italy; 9 Center of Translational and Experimental Myology, IRCCS Istituto Giannina Gaslini, Genoa, Italy; 10 Childhood Nutrition Research Group, Population, Policy and Practice Research and Teaching Department, UCL Great Ormond Street Institute of Child Health, London, UK; 11 Obesity Unit and Laboratory of Nutrition and Obesity Research, Department of Endocrine and Metabolic Diseases, IRCCS Istituto Auxologico Italiano, Milan, Italy

**Keywords:** Spinal muscular atrophy, Growth, Percentiles, Nutritional status

## Abstract

**Background:**

Spinal muscular atrophy (SMA) is a neuromuscular disorder characterized by muscle atrophy and weakness. SMA type 1 (SMA1) is the most severe form: affected infants are unable to sit unaided; SMA type 2 (SMA2) children can sit, but are not able to walk independently. The Standards of Care has improved quality of life and the increasing availability of disease-modifying treatments is progressively changing the natural history; so, the clinical assessment of nutritional status has become even more crucial. Aims of this multicenter study were to present the growth pattern of treatment-naïve SMA1 and SMA2, and to compare it with the general growth standards.

**Results:**

Body Weight (BW, kg) and Supine Length (SL, cm) were collected using a published standardized procedure. SMA-specific growth percentiles curves were developed and compared to the WHO reference data. We recruited 133 SMA1 and 82 SMA2 (48.8% females). Mean ages were 0.6 (0.4–1.6) and 4.1 (2.1–6.7) years, respectively. We present here a set of disease-specific percentiles curves of BW, SL, and BMI-for-age for girls and boys with SMA1 and SMA2. These curves show that BW is significantly lower in SMA than healthy peers, while SL is more variable. BMI is also typically lower in both sexes and at all ages.

**Conclusions:**

These data on treatment-naïve patients point toward a better understanding of growth in SMA and could be useful to improve the clinical management and to assess the efficacy of the available and forthcoming therapies not only on motor function, but also on growth.

**Supplementary Information:**

The online version contains supplementary material available at 10.1186/s13023-021-02015-9.

## Introduction

Spinal muscular atrophy (SMA) is a rare neuromuscular disorder characterized by progressive muscle atrophy, generalized weakness, and paralysis [1]. The classical form is a monogenic disorder, due to mutations in the Survival of Motor Neuron 1 (*SMN1*) gene that leads to degeneration of alpha motor neurons in the spinal cord [2]. SMA has an incidence of about 1 in 6000 to 1 in 10,000 live births [3] and is categorized into five different phenotypes based on the age of onset and the highest level of motor function achieved [4].

The most severe form compatible with life is type 1 (SMA1): SMA1 infants show the first symptoms at birth or before 6 months of age and are unable to sit unsupported [5, 6]. They are affected by the highest burden of comorbidities, including progressive bulbar dysfunction, respiratory failure and gastrointestinal symptoms, that tend to hamper their nutritional management and can lead to failure to thrive [7–10]. The onset of symptoms in SMA type 2 (SMA2) typically occurs between 7 and 18 months [11], and affected children that can achieve the ability to sit without support but are not able to walk independently. SMA2 children can show swallowing and chewing difficulties, along with respiratory problems that can require mechanical ventilation [11]. The nutritional status of the two forms has been reported to be quite the opposite: SMA1 children tend to be underweight [8, 9], while SMA2 children can be overweight or even obese [12], although undernutrition is also reported in a percentage of cases [7, 8, 12, 13].

Since the publication and implementation of the Standards of Care (SoC) for SMA in 2007 and 2018 [5, 14] there has been an improvement in prognosis and survival for patients with SMA as a result of a more proactive approach in the management of the disease and its complications [5, 14]. In addition, the increasing availability of disease-modifying treatments, including the antisense oligonucleotide nusinersen, the gene replacement therapy Onasemnogene Abeparvovec, and, more recently, the small molecule risdiplam, is progressively changing the natural history of the disease [6, 15, 16].

As body composition of children with SMA differ from those of the normal population, with SMA patients showing decreased lean body mass and increased fat mass [5, 9, 10], comparison of BMI with growth references for the general pediatric population may not reflect the nutritional status. Although the use of standard growth charts has been suggested for monitoring longitudinal growth in SMA children, the clinical assessment of their growth and nutritional status would be improved by the availability of disease-specific growth percentiles curves. This will also allow evaluation of the effects of the increasingly available novel disease-modifying treatments on growth and nutritional status.

Hence, the aim of this study is to present for the first time the growth pattern of a large sample of treatment-naïve SMA 1 and 2 children and to compare it with the reference values for the general pediatric population to clarify possible differences.

## Methods

### Participants and study design

Participants were recruited, between April 2015 and May 2018, from 5 clinical SMA referral centers in Italy (SAPRE-UONPIA, Fondazione IRCCS Ca’ Granda Ospedale Maggiore Policlinico, Milan; UO Neurologia dello Sviluppo, Fondazione IRCCS Istituto Neurologico Carlo Besta, Milan; Dipartimento di Salute della Donna e del Bambino, Università di Padova; Unità di Malattie Neuromuscolari e Neurodegenerative, Laboratorio di Medicina Molecolare, Dipartimento di Neuroscienze e Neuroriabilitazione, IRCCS Ospedale Pediatrico Bambino Gesù, Rome; IRCCS Istituto Giannina Gaslini, Genoa), all involved in a large multicenter observational study in SMA children.

Inclusion criteria were:

- Genetic confirmation of 5q-autosomal recessive SMA (either due to homozygous deletions or to compound heterozygous mutations in the SMN1 gene) [5];
- clinically confirmed diagnosis of SMA1 or SMA2 [17];
- age 0–11.99 years;
- clinical management according to the best supportive care based on the Consensus Statement for SoC in SMA [5, 17];
- absence of acute medical conditions in the 15 days before the assessment;
- not involved in any experimental pharmacological trials at the time of the assessment.

Each child underwent the anthropometric measurements [body weight (BW, kg), supine length (SL, cm)] at one of the following sites: the International Center for the Assessment of Nutritional Status (ICANS), University of Milan; Dipartimento di Salute della Donna e del Bambino, Università di Padova; Unità di Malattie Neuromuscolari e Neurodegenerative, Laboratorio di Medicina Molecolare, Dipartimento di Neuroscienze e Neuroriabilitazione, IRCCS Ospedale Pediatrico Bambino Gesù, Rome; Dipartimento di Neuroscienze e Riabilitazione, IRCCS Istituto Giannina Gaslini, Genoa.

The study was approved by the Ethics Committee of the University of Milan (n.7/16), accepted by the other institutional partners and complied following the Helsinki declaration. The parents, on behalf of their children, gave their written informed consent to the study.

### Study variables

Demographic, clinical, and anthropometric variables were collected. The demographic variables included age at study date and sex. Clinical variables included type of breathing: spontaneous compared with mechanical ventilation (non-invasive mechanical ventilation or invasive ventilation-tracheostomy), type of feeding (oral compared with nasogastric tube or gastrostomy). The clinical variables were collected by pediatric neurologists (GiBa, CA, AD, MP, CB) 1 day before nutritional assessment.

### Anthropometric measurements

In each center, all anthropometric measurements were collected by the same dieticians who attended a 1-day training workshop and used standardized measuring procedures [18]. The measurements were taken in a standard setting at the same time, with the child undressed and in the fasting state.

According to the WHO child growth standards, BW was collected to the nearest 0.1 kg with an electronic wheelchair scale accurate to 0.1 kg (Seca 664, Seca GmbH, Hamburg, Germany). The guardian stood alone on the scale while the examiner clicked the tare button on the scale. This set the scale readout to zero. The child was then handed to the adult on the scale. In this way the scale recorded only the child’s weight [19].

SL was measured by a non-elastic tape (Gima 27341, Gima S.p.A., Gessate, Italy) to the nearest 0.1 cm on the child’s right side. Dietitian and caregivers positioned the child supine on an appropriate exam table with the Frankfort plane perpendicular to the table (support), shoulders and buttocks resting against the table, arms along the trunk, palms facing up, legs as straight as possible and in contact with the table (board). In cases of scoliosis and contractures, segmental lengths were taken three times from the top of the head to the greater trochanter of the hip, from the hip to the femoral epicondyle of the knee, and from the knee to the distal point of the calcaneus, were then added and the total mean measurement was recorded [13].

Body mass index (BMI) was calculated by the following formula: BW (kg)/SL^2^ (m^2^).

BW, BL, and BMI z-scores were derived using the WHO Growth Charts [20]

A z-score < − 1 was considered under the normal range, between the − 1 and + 1 was considered normal, between the + 1 and + 2 was considered indicative of overweight, and a z-score > + 2 was considered indicative of obesity [20].

### Statistical analysis

Most continuous variables were not normally distributed, and all are reported as median and interquartile range (IQR, 25th–75th percentile). Discrete variables are reported as the number and proportion of subjects with the characteristic of interest. The median values of anthropometric variables were compared between spontaneously breathing with mechanical ventilation children and mouth-fed and children with artificial feeding using the Wilcoxon Rank sum test. A value of *p* < 0.05 was considered statistically significant. Statistical analysis was performed using IBM SPSS Statistics software version 26.0 for Windows (IBM, Armonk, NY, USA).

Derived SMA-specific growth percentiles curves were developed for SMA1 and SMA2 patients using the LMS method [21]. Sex-specific values by month of age were obtained for all anthropometric outcomes using the LMS method (LMS Chart Maker, Medical Research Council, UK) [21]. This statistical approach, widely used to construct reference data for traits which incorporate the effects of growth, provides three outputs: (a) a smoothed median (M or mu) curve which represents how the outcome varies in relation to age; (b) the coefficient of variation (S or sigma), which models the scatter of values around the mean and adjusts for any non-uniform dispersion; and (c) the skewness (L or lambda) which is addressed using age-specific Box–Cox transformation to achieve a normal distribution. The program also calculates centile values by age. BMI was fitted using original age, and weight and height using re-scaled age, which improves the goodness of fit for monotonic data by fitting the M curve twice. Goodness-of-fit was assessed with the Bayesian Information Criterion, adding an extra unit of complexity to the model only if it reduced the deviance by more than log_e_(N) units, where N is the sample size.

To compare these growth percentiles curves against WHO reference data, we superimposed centiles over the age range 0–5 years for the SMA1 patients, and 0–10 years for the SMA2 patients. We plotted the 10th, 25th, 50th, 75th and 90th centiles for WHO, and the 10th, 25th, 50th, 75th and 90th centiles for the patient data. Further comparison between WHO-scores of SMA patients and the median of the reference population was accomplished by using one-sample t tests. Data are reported as mean and confidence intervals (CI). A *p* value < 0.05 was considered statistically significant.

## Results

We recruited 133 SMA1 children (56.4% females and 43.6% males) and 82 SMA2 children (48.8% females and 51.2% males) (Additional file 1: Fig. S1). Median ages were 0.6 (IQR: 0.4–1.6) and 4.1 (IQR: 2.1–6.7) years, respectively. Table 1 shows the distributions of demographic, clinical (ventilation, feeding) and anthropometric variables.

**Table 1:** Demographic, clinical and anthropometric variables in SMA1 and SMA2 children

	SMA1			SMA2		
	Male	Female	Total	Male	Female	Total
	N = 58 (43.6%)	N = 75 (56.4%)	N = 133	N = 42 (51.2%)	N = 40 (48.8%)	N = 82
*Demographic variables*						
Age (years)	0.7 (0.4; 2.1)	0.6 (0.4; 1.4)	0.6 (0.4; 1.6)	4.3 (2.2; 7.0)	3.9 (1.9; 6.7)	4.1 (2.1; 6.7)
Age classes						
0–1 year	35 (60.3%)	51 (68.0%)	86 (64.7%)	1 (2.4%)	0 (0.0%)	1 (1.2%)
1–3 years	14 (24.1%)	10 (13.3%)	24 (18.0%)	12 (28.6%)	15 (37.5%)	27 (32.9%)
3–6 years	4 (6.9%)	8 (10.7%)	12 (9.0%)	15 (35.7%)	13 (32.5%)	28 (34.1%)
6–10 years	3 (5.2%)	4 (5.3%)	7 (5.3%)	9 (21.4%)	10 (25.0%)	18 (22.0%)
> 10 years	2 (3.5%)	2 (2.7%)	4 (3.0%)	5 (11.9%)	2 (5.0%)	8 (9.8%)
*Clinical variables*						
Ventilation						
Spontaneous breathing	58 (63.8%)	47 (62.7%)	85 (63.3%)	31 (73.8%)	26 (65.0%)	57 (69.5%)
Non invasive-ventilation	13 (22.4%)	23 (30.7%)	36 (27.1%)	11 (26.2%)	14 (35.0%)	25 (30.5%)
Tracheostomy	8 (13.8%)	5 (6.6%)	13 (9.6%)	0 (0.0%)	0 (0.0%)	0 (0.0%)
Total mechanical ventilation	21 (36.2%)	28 (37.3%)	49 (36.7%)	11 (26.2%)	14 (35.0%)	25 (30.5%)
Feeding						
By mouth	48 (82.8%)	57 (76.0%)	105 (79.0%)	42 (100.0%)	40 (100.0%)	82 (100.0%)
Nasogastric tube	1 (1.7%)	5 (6.7%)	6 (4.5%)	0 (0.0%)	0 (0.0%)	0 (0.0%)
Percutaneous endoscopic gastrostomy	9 (15.5%)	13 (17.3%)	22 (16.5%)	0 (0.0%)	0 (0.0%)	0 (0.0%)
Total artificial feeding	10 (17.2%)	18 (24.0%)	28 (21.0%)	0 (0.0%)	0 (0.0%)	0 (0.0%)
*Anthropometric variables*						
Weight (kg)	7.2 (6.2; 9.1)	7.4 (6.2; 9.3)	7.4 (6.2; 9.2)	14.4 (11.5; 19.2)	13.6 (10.4; 20.0)	14.2 (10.7; 19.8)
Weight z-score (WHO)	− 1.64 (− 2.39; − 0.69)	− 0.72 (− 1.73; − 0.05)	− 1.01 (− 2.17; − 0.12)*^δ^	− 1.04 (− 2.26; − 0.24)	− 1.06 (− 2.17; − 0.36)	− 1.04 (− 2.26; − 0.33)*
Length (m)	0.72 (0.67; 0.86)	0.72 (0.66; 0.82)	0.72 (0.66; 0.84)	1.05 (0.89; 1.17)	0.97 (0.84; 1.11)	1.02 (0.87; 1.15)
Length z-score (WHO)	0.54 (− 0.28; 1.50)	1.03 (− 0.17; 1.99)	0.82 (− 0.15; 1.80)	− 0.17 (− 0.78;1.20)	− 0.62 (− 2.12; 0.69)	0.41 (− 1.78; 0.83)
BMI (kg/m^2^)	13.4 (12.1; 14.9)	13.9 (12.7; 14.9)	13.7 (12.3; 14.9)	14.1 (12.6; 15.9)	14.1 (13.2; 15.9)	14.1 (12.8; 15.9)
BMI z-score (WHO)	− 2.61 (− 3.87; − 1.72)	− 2.22 (− 3.19; − 1.08)	− 2.48 (− 4.17; − 1.43)*^δ^	− 1.23 (− 2.91; − 0.35)	− 0.75 (− 1.93; − 0.45)	− 1.28 (− 2.61; − 0.13)*

Data are presented as median and interquartile range (IQR) for continuous measures, and n (%) for categorical measures

*BMI* body mass index (Body Weight (kg)/Body Length (m^2^))

*p < 0.050 Spontaneous breathing versus mechanical ventilation

^δ^*p* < 0.050 Fed by mouth versus total artificial feeding

In SMA1 children, both BW and BMI z-scores were significantly lower in mechanically ventilated compared to spontaneously breathing patients (− 1.8 vs. − 1.0, *p* = 0.003; − 3.1 vs. − 2.1, *p* ≤ 0.001; respectively), or in tube-fed compared to orally-fed patients (− 1.2 vs. − 2.2, *p* = 0.012; − 3.4 vs. − 2.2; *p* = 0.026; respectively). Similarly, in SMA2 children, both BW and BMI z-score were significantly lower in the mechanically ventilated patients compared to those on spontaneous breathing (− 1.8 vs. − 0.9, *p* = 0.003; − 2.9 vs. − 0.8, *p* = 0.019; respectively).

### SMA1 growth patterns

Additional file 2: Fig. S2 shows smoothed percentile curves of weight, supine length, and BMI-for-age for girls and boys with SMA1. Additional file 3: Table S1 shows the LMS values and percentile distributions for weight, supine length, and BMI-for-age of SMA1 patients.

Weight and supine length growth are more linear in SMA1 girls compared to SMA1 boys, who show a decrease in weight, length, and BMI velocity at about 3 years of age.

### SMA2 growth patterns

Additional file 4: Fig. S3 shows smoothed percentile curves of weight, supine length, and BMI-for-age for girls and boys with SMA2. Additional file 5: Table S2 shows the LMS values and percentile distributions for weight, supine length, and BMI-for-age of SMA1 patients.

SMA2 girls have a linear weight and length growth velocity, while SMA2 boys show a decrease in both weight and length velocity since about 6 years of age.

### Comparisons between WHO and SMA growth percentiles

**Fig. 1:**
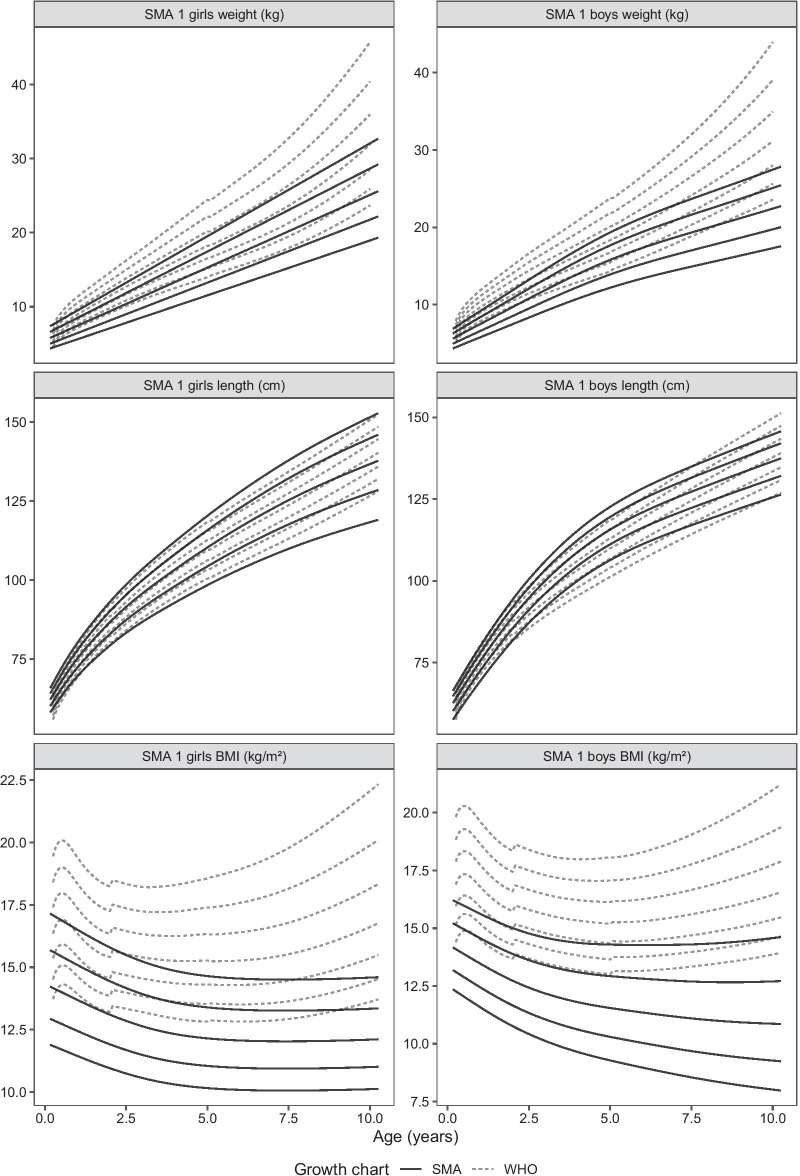
Comparisons of body weight, supine length, and BMI-for-age between SMA1 patients and WHO growth percentiles (10th, 25th, 50th, 75th and 90th)

**Fig. 2:**
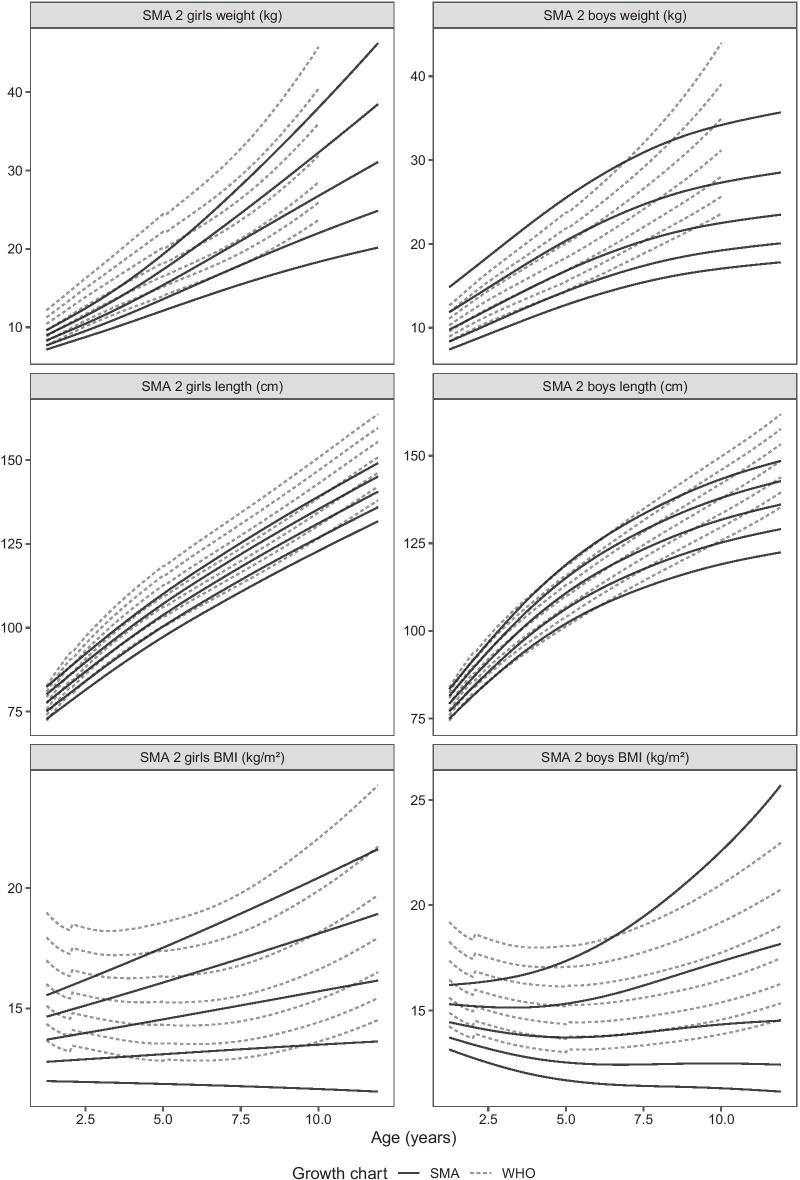
Comparisons of body weight, supine length, and BMI-for-age between SMA2 patients and WHO growth percentiles (10th, 25th, 50th, 75th and 90th)

Figures 1 and 2 show graphical comparisons of body weight, supine length, and BMI-for-age between WHO growth percentiles and SMA1 and SMA2 patients, respectively.

In SMA1, weight is significantly lower than healthy peers in both sexes and at all age classes [mean WHO z-score − 1.334 (CI − 1.608 to − 1.071); *p* ≤ 0.001], while supine length is more variable. SMA1 girls are longer than their healthy peers up to the age of 1 year [mean WHO z-score 1.02 (CI 0.550–1.491); *p* ≤ 0.001], and then they follow more closely the growth pattern of the general pediatric population [mean WHO z-score 0.049 (CI − 0.556 to 0.656); *p* = 0.869]. Supine length is similar to the general pediatric population in boys in all age classes [mean WHO z-score 0.313 (CI − 0.161 to 0.787); *p* = 0.192]. BMI is lower for both sexes in all age classes (mean WHO z-score − 2.339; CI − 2.643 to − 2.034; *p* < 0.001).

In SMA2, weight is generally lower in both sexes up to 6 years (mean WHO z-score − 1.084 (CI − 1.419 to − 0.749); *p* ≤ 0.001), when it starts to follow the growth pattern of the general pediatric population [mean WHO z-score − 0.663 (CI − 1.544 to 0.219); *p* = 0.132]. There is a small proportion of boys who are heavier [5 patients, 6% of SMA2 sample; mean WHO z-score 1.654 (CI 1.543–1.906)]. Supine length is similar to the general pediatric population in boys [mean WHO z-score − 0.292; (CI − 0.792 to 0.208); *p* = 0.244] and girls [mean WHO z-score − 0.531 (CI − 1.616 to 0.555); *p* = 0.310], but shorter in girls from 3 years [mean WHO z-score − 0.982 (CI − 1.421 to − 0.543); *p* ≤ 0.001]. BMI is also typically lower in both sexes up to 6 years [mean WHO z-score − 1.262 (CI − 1.666 to − 0.857); *p* = 0.000] and similar to the general pediatric population afterwards [mean WHO z-score − 0.388 (CI − 1.515 to 0.738); *p* = 0.481], except for boys which are heavier at older ages [mean WHO z-score 1.534 (CI 1.293–1.902); *p* = 0.048; mean age 7 (5.0–9.3) years].

A Z-score calculator and printable PDFs of SMA growth percentiles curves can be used as a further aid (https://jscalc.io/calc/Q91zp6clkwI9PVBn).

## Discussion

This study provides, for the first time, growth curves derived from a large, well-characterized sample of treatment-naïve SMA1 and SMA2 children. These data were collected as part of a large natural history cohort study in 5 Italian centers, where patients were regularly monitored and followed up according to the SoC for SMA [5, 14], and using a standardized and validated protocol of anthropometric measures on SMA [18]. Over the past decade, the implementation of SoC in the management of patients with SMA has strongly improved their survival and quality of life. Additionally, the approval of disease-modifying treatments by the US Food and Drug Administration and the European Medicines Agency, and their increasing availability to patients in many countries are gradually changing the natural history of the disease [15, 22]. For this reason, the availability of reference data in treatment-naïve patients can be particularly helpful and timely to assess the efficacy of the available and forthcoming therapies not only on motor function and survival, but also on other domains, including growth and nutritional status [23].

To date, few studies have investigated the nutritional aspects in SMA [8, 13, 24–27], all of them being on small samples and few using a standardized protocol to assess growth pattern [28]. When compared to WHO percentiles, the present study confirms that in SMA1 weight is significantly lower than healthy peers in both sexes and at all ages, as consistently reported in previous studies [8, 13]. Although the numbers were limited to split the sample into different categories of severity based on the clinical presentation (symptoms in the first 2 weeks of life [type 1A], within the third month [type 1B], and between 3 and 6 months [type 1C]) [29, 30], our data show that patients at the most severe end of the spectrum, requiring respiratory and nutritional support, are those who had lower weight. Importantly, SMA2 patients have lower weight than the general pediatric population since the first years of life, and those requiring non-invasive ventilatory support are also those having lower weight. These differences should be taken into consideration when monitoring nutritional status in SMA children, as they can be helpful in guiding and customizing early nutritional intervention in this population. Of note, we found that SMA1 children had an above average SL compared to the general pediatric population, while BMI tended to be significantly lower, consistent with the weight pattern. This could be due to the fact that we used segmental length because of the possible presence of contractions, inability to stand, scoliosis and other musculo-skeletal deformities, and this may have resulted in overestimates, despite having used a standardized method [13, 18].

Our findings confirm that BMI references based on the general pediatric population are not good indicators of the nutritional status in SMA children as they show a growth pattern which is specific to the condition. This is further supported by previous studies by us and others showing that fat mass is increased, and total fat-free mass and lean mass decreased in both SMA1 and SMA2 compared to healthy peers, with fat mass increasing with age [10, 12, 13, 25–27]. Furthermore, we have also shown that body composition mildly to moderately correlates with motor function depending on the clinical phenotype [10]. The risk of sub-optimal nutritional management in children with SMA, with adverse effects on their body composition and nutritional status and ultimately their functional abilities, can be at least partly overcome using disease-specific growth percentiles curves.

Interestingly, we have also identified the presence of gender differences in the growth pattern of SMA children: weight and supine length growth are more linear in girls compared to boys, with SMA1 boys having a decrease in weight, length, and BMI velocity, and a small proportion of SMA2 boys (6% of our SMA2 sample) showing constantly a higher body weight then the general pediatric population. In an animal model, SMA females displayed better therapeutic outcomes than SMA male mice and a better functional response [28], but further studies are needed to better understand the sex-dependent influence on disease progression, pathophysiology, and response to treatment [28]. Our findings further highlight the role that gender plays in determining sex-specific variations and vulnerabilities in SMA and may have important clinical implications to customize the nutritional management since diagnosis.

This study has several strengths. The age distribution of the sample allowed us to have cross-sectional data in treatment-naïve patients. This could be crucial to potentially investigate the effect of drugs on all the collected parameters in different age groups. Secondly, this is the largest work in the literature studying nutritional status in children with a confirmed diagnosis of SMA. The third strength is the data quality: these data were collected prospectively through a standardized protocol [17], and not retrospectively from chart reviews; dispersion of patients was overcome by strict cooperation between the involved centers that also allowed an equal distribution in the different classes of age. Moreover, we have developed easily accessible and printable percentiles curves that could be implemented in the clinical practice.

A possible limitation of the study is that all children were Caucasian, and their patterns of nutritional status may not be globally representative of all ethnic groups. Furthermore, as this is a cross-sectional and not a longitudinal study, we could not investigate the growth patterns of individual SMA children: the sample size was not large enough to build a model of growth, in spontaneously breathing and mouth-fed children and in children with mechanical ventilation and/or tube feeding. Moreover, due to the low number of breastfed children (6% of total sample), the different types of feeding (breastfeeding, formula feeding or mixed feeding) were also not taken into account despite it is well documented that the growth patterns of breast-fed and formula-fed infants differ significantly [31, 32]. The clinical diagnosis of SMA was made in accordance with the first version of the SoC recommendations [16], because the recruitment took place between April 2015 and May 2018: for this reason, the percentiles were divided by SMA1 and SMA2 and not according to the functional classification reported in the 2018 update of the SoC recommendations, which classifies SMA patients as sitters and non-sitters; also, *SMN2* copy number was not systematically collected in our study. Further studies in children receiving disease-modifying treatments will allow comparisons also based on the motor functional classification and *SMN2* copy number. Moreover, how spine and joint deformity could potentially affect growth pattern will require further investigation in future studies. Finally, the number of growth measurements was small at the older ages, which may limit the precision of the estimates at those ages.

Key questions still remain unanswered and will require further investigations in the future: (1) how the new disease-modifying treatments will impact the nutritional status of children with SMA by promoting the development of new phenotypes with preserved swallowing and respiratory function, particularly when treatment is administered at a pre-symptomatic or early post-symptomatic phase; (2) how weight and height influence other aspects of the disease, including general health, respiratory status, cardiac and metabolic status, risk for fractures and/or scoliosis; (3) how feeding type (breastfeeding, formula, mixed feeding), demographic and socioeconomical status (urban and rural areas, medical level, economic level, family care, education, etc.) could affect growth pattern in SMA disease.

## Conclusion

Nutritional aspects play a significant role in the multidisciplinary management of children with SMA1 and SMA2, particularly in the treatment of nutritional derangements including swallowing and gastrointestinal problems. The increase in the knowledge on nutritional aspects of patients with SMA is crucial for the appropriate management of patients. However, the lack of specific, standardized and coordinated nutritional assessment for SMA patients [28] is common and the use of reference data [20] developed for healthy children increases the risk of inadequate nutritional support because of the peculiar nature of SMA [5, 9].

These data on treatment-naïve patients point toward a better understanding of the natural progression of the disease, to improve the clinical and nutritional management of patients and to evaluate the effects of disease-modifying treatments on growth in SMA1 and SMA2.

## Supplementary Information

**Additional file 1: Fig. S1.** Participant flow chart.

**Additional file 2: Fig. S2.** Body weight, supine length, and BMI-for-age percentile curves (10th, 25th, 50th, 75th and 90th) of SMA1 patients.

**Additional file 3: Table S1.** Observed means, SD and percentile distributions for weight, supine length, and BMI-for-age of SMA1 patients.

**Additional file 4: Figure S3.** Body weight, supine length, and BMI-for-age percentile curves (10th, 25th, 50th, 75th and 90th) of SMA2 patients.

**Additional file 5: Table S2.** Observed means, SD and percentile distributions for weight, supine length, and BMI-for-age of SMA2 patients.

## Data Availability

The corresponding authors will cooperate with any reasonable requests from the journal for data or additional information should questions about the paper arise after publication.

## Abbreviations

BMI: Body mass index
BW: Body weight
CI: Confidence interval
SL: Supine length
SMA: Spinal muscular atrophy
SMA1: Spinal muscular atrophy type 1
SMA2: Spinal muscular atrophy type 2
SMN1: Survival of motor neuron 1
SoC: Standard of care
WHO: World Health Organization
