# Supplementary material for: Growth patterns in children with spinal muscular atrophy
Source: Orphanet J Rare Dis. 2021 Sep 4;16:375. doi: 10.1186/s13023-021-02015-9 (PMC8418717; doi:10.1186/s13023-021-02015-9)

Weight-for-age SMA 1 GIRLS

2 months to 10 years

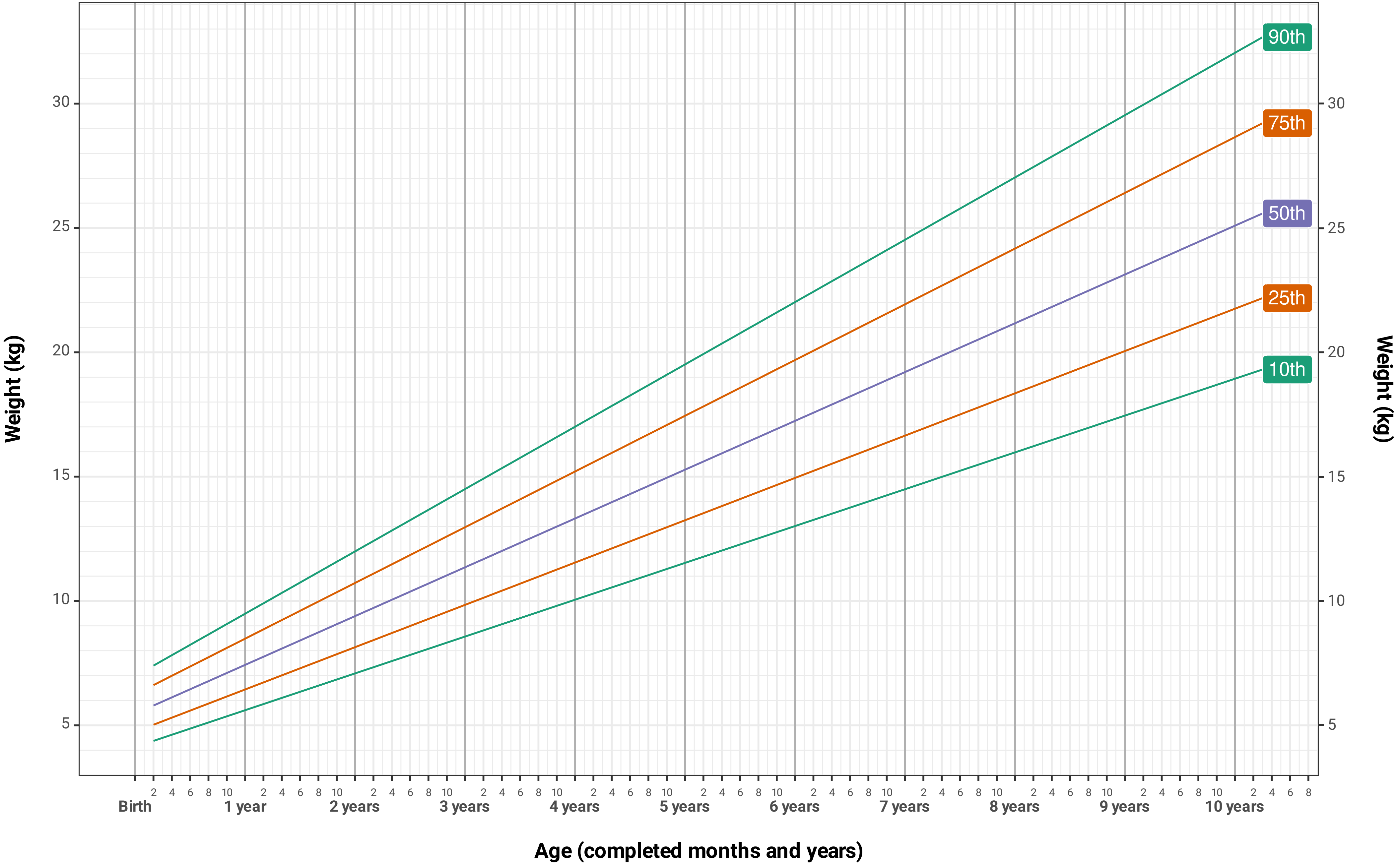

Weight-for-age SMA 1 BOYS

2 months to 10 years

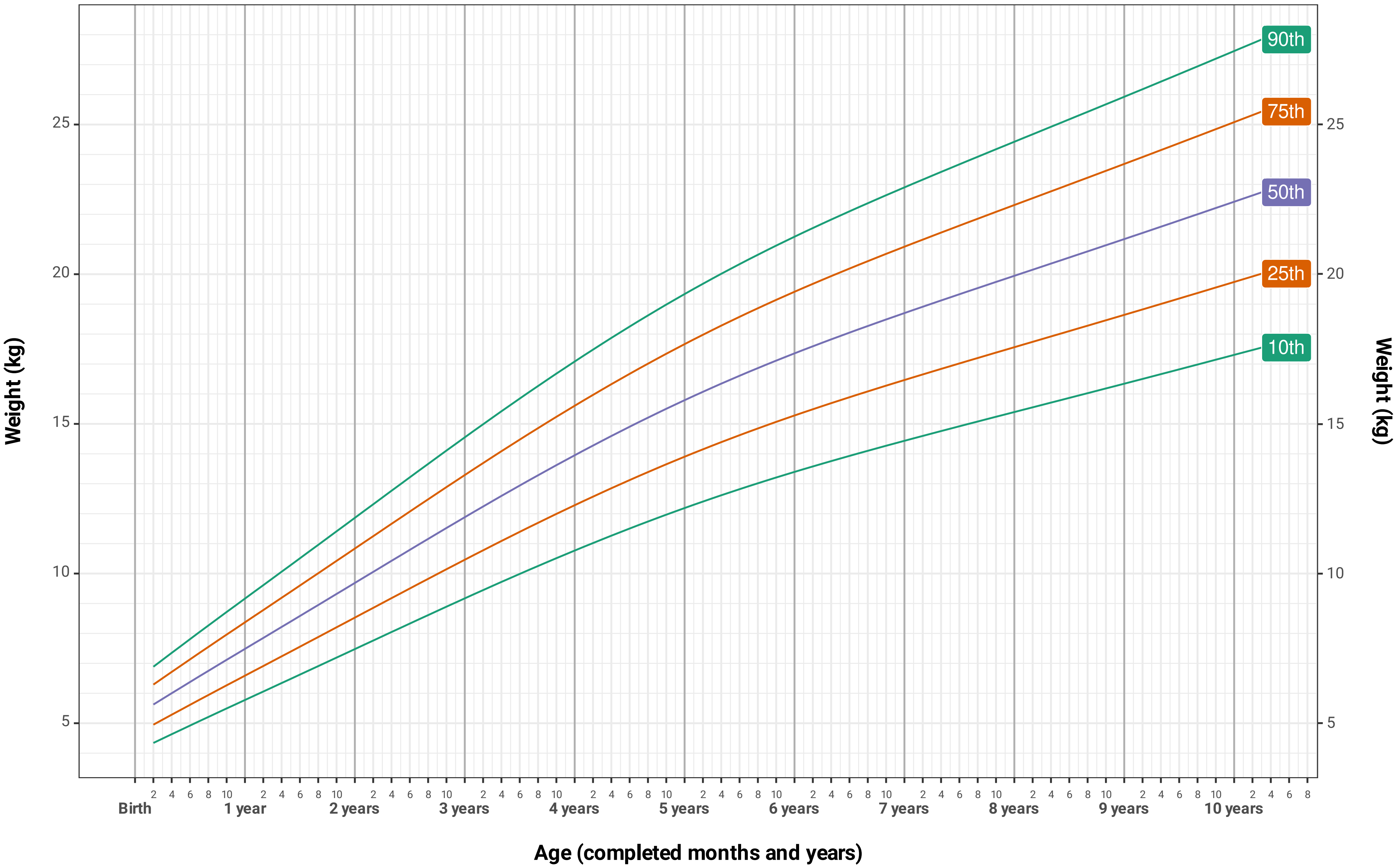

Length-for-age SMA 1 GIRLS

2 months to 10 years

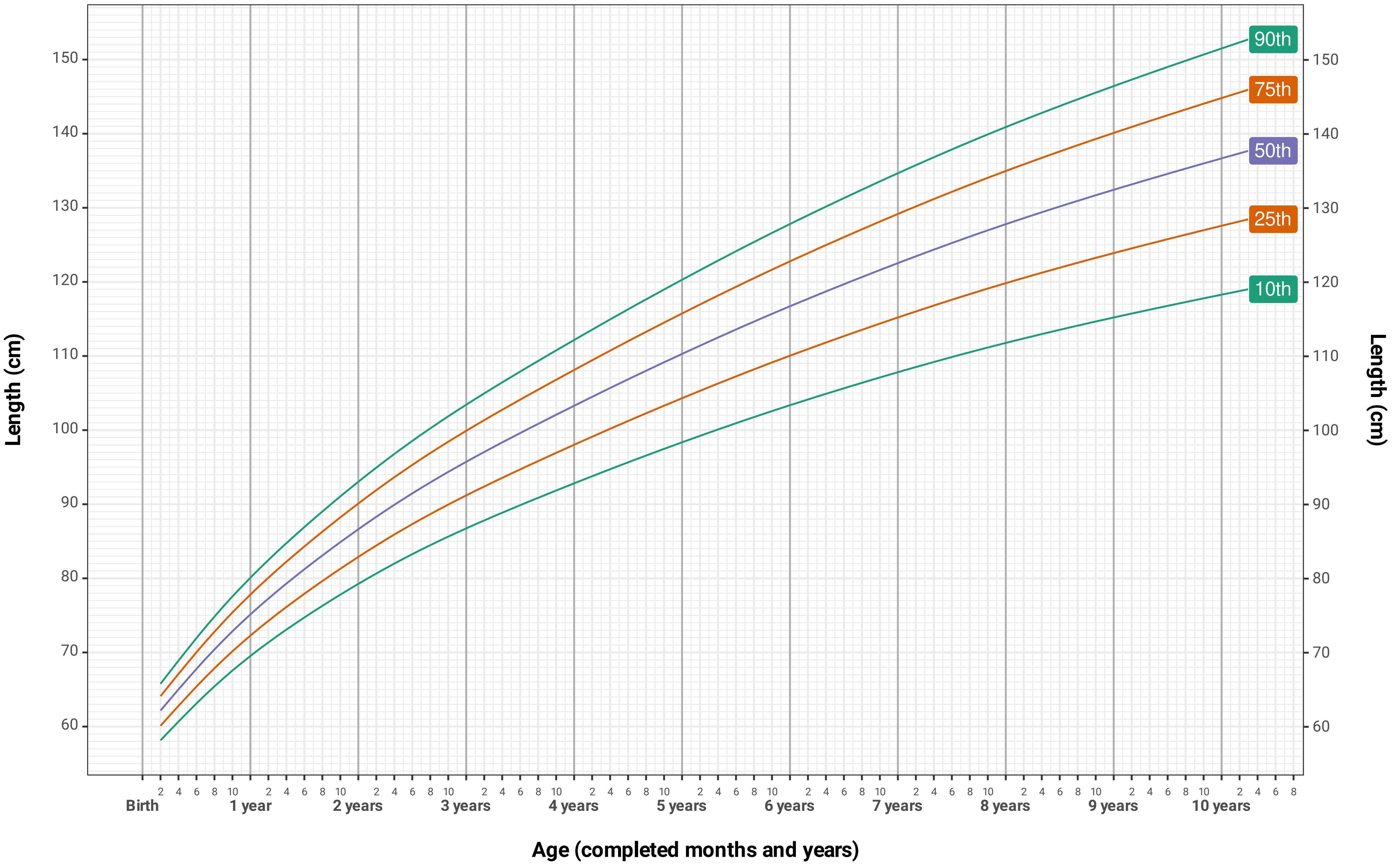

Length-for-age SMA 1 BOYS

2 months to 10 years

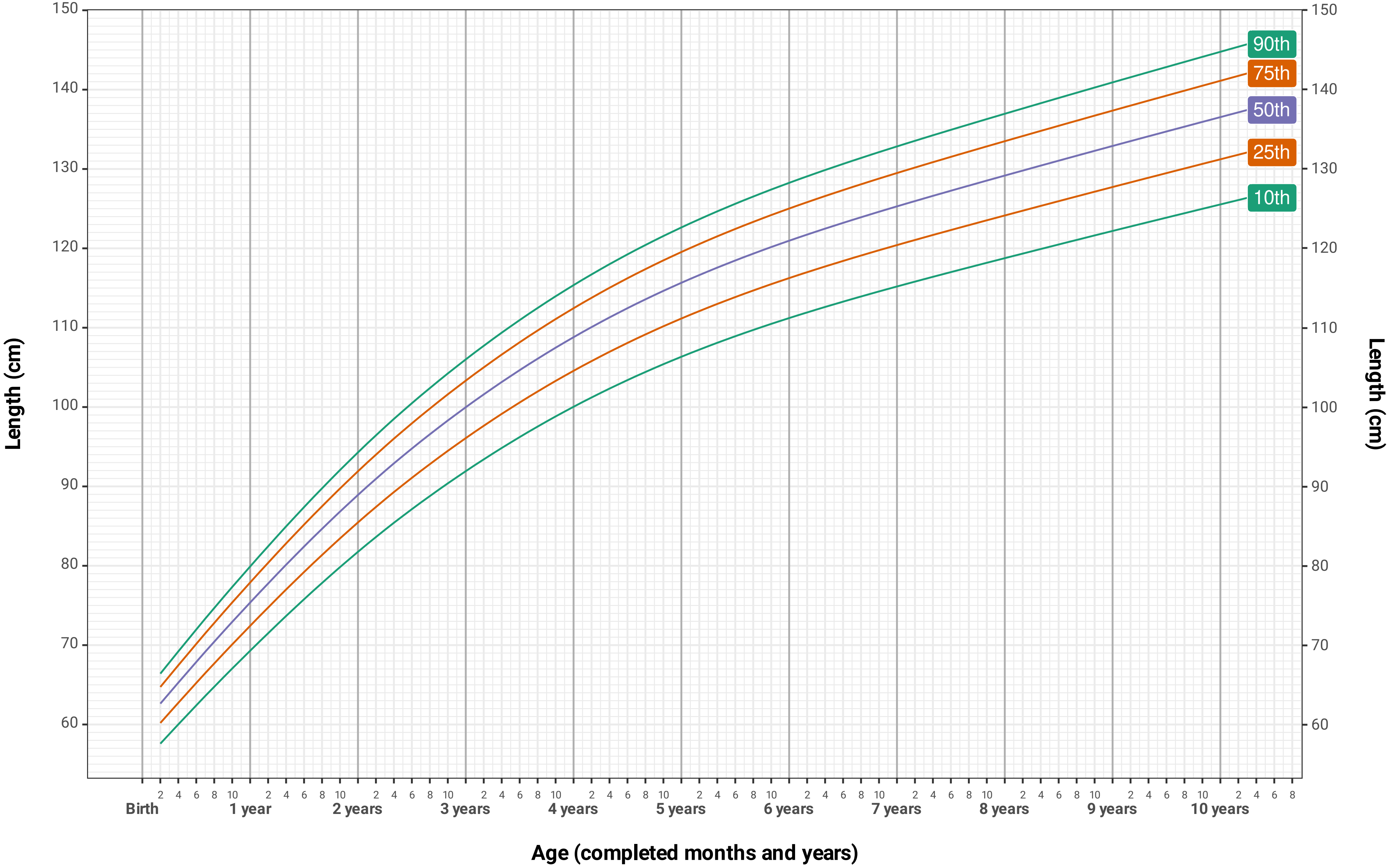

BMI-for-age SMA 1 GIRLS

2 months to 10 years

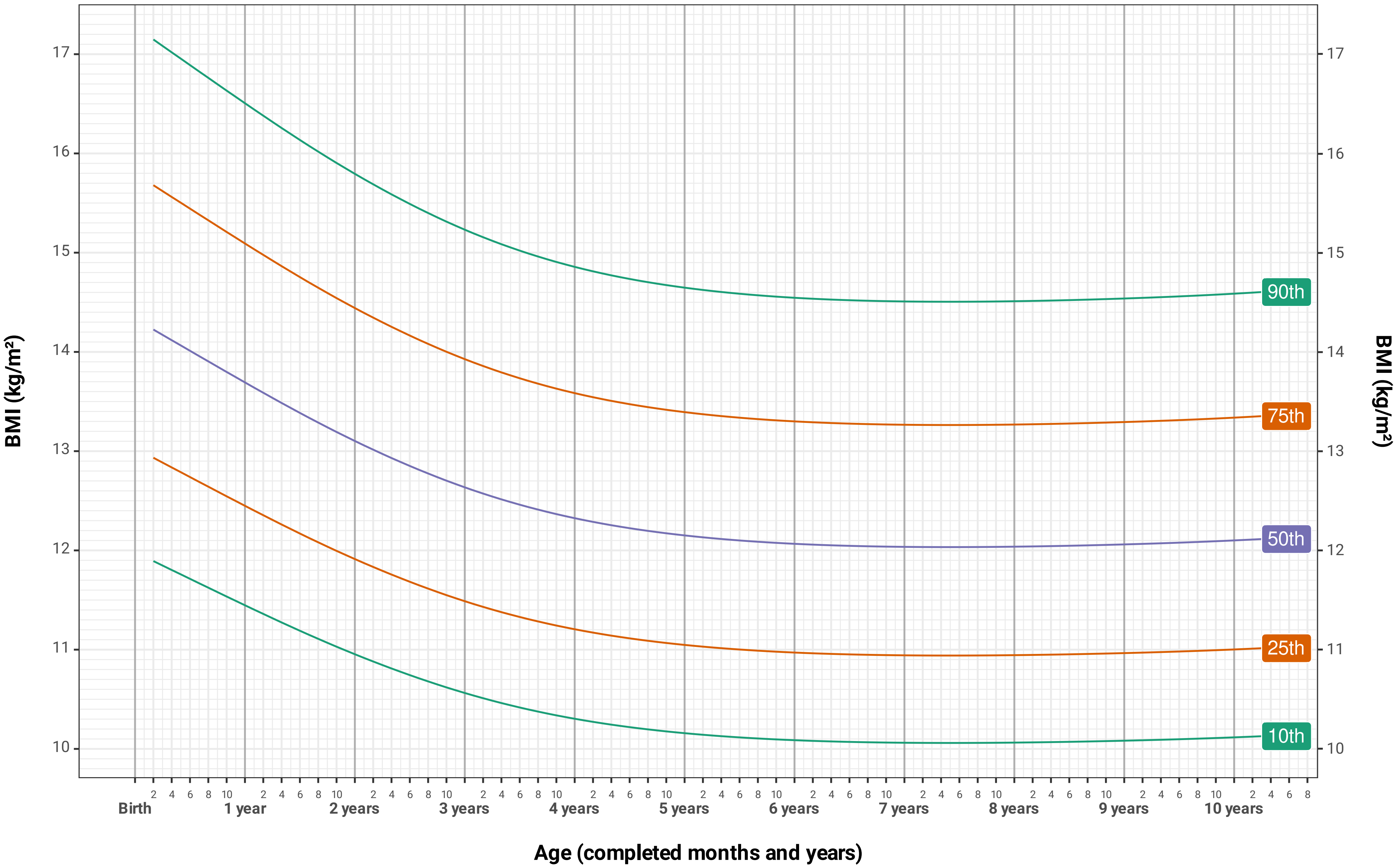

BMI-for-age SMA 1 BOYS

2 months to 10 years

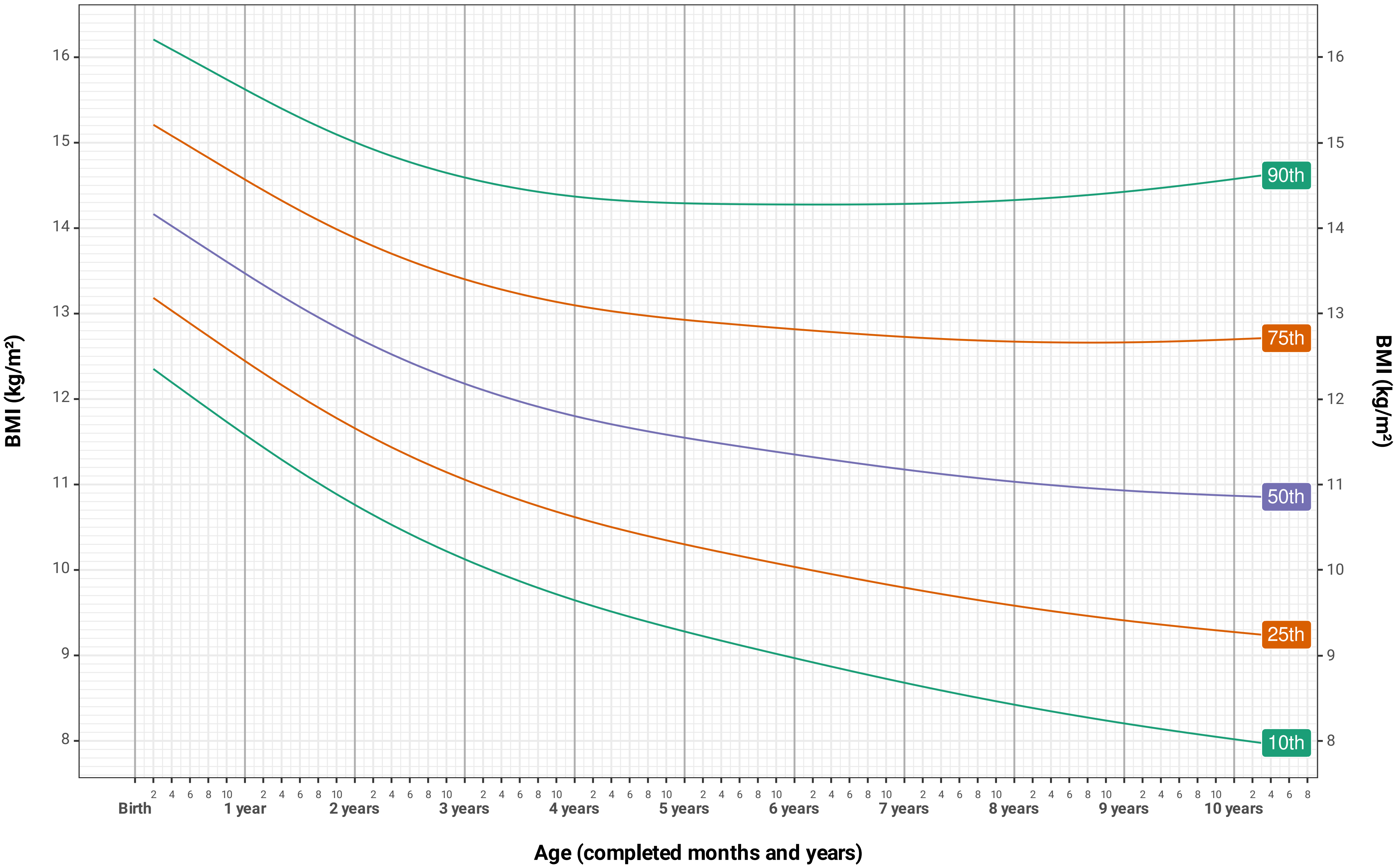

Supplement: Supplementary file 2 — Additional file 2: Fig. S2. Body weight, supine length, and BMI-for-age percentile curves (10th, 25th, 50th, 75th and 90th) of SMA1 patients. [file 13023_2021_2015_MOESM2_ESM.pdf]
